# Identification of a Small Molecule That Modifies MglA/SspA Interaction and Impairs Intramacrophage Survival of *Francisella tularensis*

**DOI:** 10.1371/journal.pone.0054498

**Published:** 2013-01-23

**Authors:** Algevis P. Wrench, Christopher L. Gardner, Claudio F. Gonzalez, Graciela L. Lorca

**Affiliations:** Department of Microbiology and Cell Science, Genetics Institute, Institute of Food and Agricultural Sciences, University of Florida, Gainesville, Florida, United States of America; Centre National de la Recherche Scientifique, Aix-Marseille Université, France

## Abstract

The transcription factors MglA and SspA of *Francisella tularensis* form a heterodimer complex and interact with the RNA polymerase to regulate the expression of the *Francisella* pathogenicity island (FPI) genes. These genes are essential for this pathogen’s virulence and survival within host cells. In this study, we used a small molecule screening to identify quinacrine as a thermal stabilizing compound for *F. tularensis* SCHU S4 MglA and SspA. A bacterial two-hybrid system was used to analyze the *in vivo* effect of quinacrine on the heterodimer complex. The results show that quinacrine affects the interaction between MglA and SspA, indicated by decreased β-galactosidase activity. Further *in vitro* analyses, using size exclusion chromatography, indicated that quinacrine does not disrupt the heterodimer formation, however, changes in the alpha helix content were confirmed by circular dichroism. Structure-guided site-directed mutagenesis experiments indicated that quinacrine makes contact with amino acid residues Y63 in MglA, and K97 in SspA, both located in the “cleft” of the interacting surfaces. In *F. tularensis* subsp. *novicida*, quinacrine decreased the transcription of the FPI genes, *iglA, iglD, pdpD* and *pdpA*. As a consequence, the intramacrophage survival capabilities of the bacteria were affected. These results support use of the MglA/SspA interacting surface, and quinacrine’s chemical scaffold, for the design of high affinity molecules that will function as therapeutics for the treatment of Tularemia.

## Introduction

Transcriptional control is a key factor in the regulation of virulence gene expression, in nearly all known bacterial pathogens. Although many mechanisms of such regulation are known, the major control point by far is transcription initiation, where transcription factors interact with RNA polymerase (RNAP) to modulate its recruitment to specific gene promoters. Members of the stringent starvation protein A (SspA) family are transcription regulators that interact with RNAP, to modulate the expression of genes required for both pathogenesis and survival during stationary phase-induced stress [1]–[3]. Many pathogens encode for proteins orthologous to SspA, including *Yersinia pestis*, *Neisseria gonorrhoeae*, *Francisella tularensis* and *Vibrio cholerae* [4]–[7]. In *Francisella tularensis*, however, a unique mechanism of interaction occurs, where two SspA protein members (annotated as SspA and MglA) form a heterodimer complex and bind to RNAP [8]. Furthermore, additional transcriptional factors make contact with the MglA/SspA/RNAP complex, to alter gene expression and promote pathogenesis. These include the putative DNA binding protein PigR (FevR) and the response regulator PmrA [9]–[11]. These protein-protein interactions positively regulate the expression of genes clustered in the *Francisella* pathogenicity island (FPI), which are required for virulence and intracellular growth [12]–[15]. Currently, scarce information is available on environmental signals that modulate the interplay between DNA binding transcription factors and the MglA/SspA complex. It has been determined that genes under the control of the Mgl/SspA complex are involved in oxidative stress responses [16]. Genetic data has indicated that *F. tularensis* mutants that do not synthesize the alarmone ppGpp are impaired in PigR interactions with the MglA/SspA complex affecting the virulence gene expression [9]. However, direct binding of the small molecule to any of the proteins has yet to be established.

The intracellular pathogen *F. tularensis* is the causative agent of tularemia, a zoonotic disease affecting humans and small mammals [17]–[19]. Due to its high level of infectivity and lethality, *F. tularensis* is considered a viable bioterrorism agent [20], [21]. Currently, Tularemia can be treated with antibiotics such as streptomycin and gentamicin [22]. However, the identification of new therapeutics is very significant, since *Francisella* can easily be genetically modified and therefore its sensitivity to known antibiotics could be compromised [23]–[25].

The manipulation of protein-protein interactions, as targets for therapeutics, is a new and expanding field of research [26]–[29]. In this regard, the *Francisella* MglA/SspA complex is a very attractive system that offers at least three usable interactions: i) with each other, ii) with the RNAP, and iii) with the DNA binding transcription factors FevR (PigR) and PmrA. Each of these interactions could potentially be modulated by the action of small molecules. In fact, it was reported that the levels of ppGpp modulate the activity of PigR (FevR) and its interactions with MglA/SspA/RNAP complex *in vivo* [9]. However, no evidence is available to confirm that the effect is due to a direct interaction with the FevR (PigR) regulator, the MglA/SspA heterodimer, the RNAP, or due to altered levels of an unknown intracellular metabolite.

Here we report the identification of a small molecule that specifically modified MglA and SspA interactions *in vitro* and *in vivo*. Using structure guided site directed mutagenesis, we were able to determine that quinacrine hydrochloride (referred to as quinacrine) binds in the “cleft region” formed by the MglA/SspA heterodimer. The biochemical evidence provided herein suggests that a biologically relevant molecule may act to modulate the expression of pathogenicity determinants in *F. novicida,* and provide the putative binding residues for such interactions.

## Results

### Identification of Small Molecules that Increase MglA and SspA Thermal Stability

To identify small molecules that may modify the MglA/SspA heterodimer interactions, a small molecule screen was performed using the Prestwick chemical library, by differential scanning fluorometry [30], [31]. To this end, the *mglA* and *sspA* genes from *F. tularensis* SCHU S4 were cloned, and the proteins purified. The *E. coli* SspA protein was also included in this study, since it has previously been physiologically and structurally characterized. The *F. tularensis* SspA (Ft-SspA) was obtained at very low concentrations (yield = 2±0.05 mg/L) when expressed individually, while MglA (Ft-MglA) and *E. coli* SspA (Ec-SspA) were soluble (yield = 9±0.1 g/L and 11±0.2 mg/L, respectively). Interestingly, Ft-SspA could be co-purified in the presence of Ft-MglA with a yield of 11±0.1 mg/L, indicating that the strong interaction between MglA and SspA improves the solubility of Ft-SspA. Based on these results, the Ec-SspA, Ft-MglA and the Ft-MglA/Ft-SspA complex were chosen to test the effect of small molecules.

**Figure 1:**
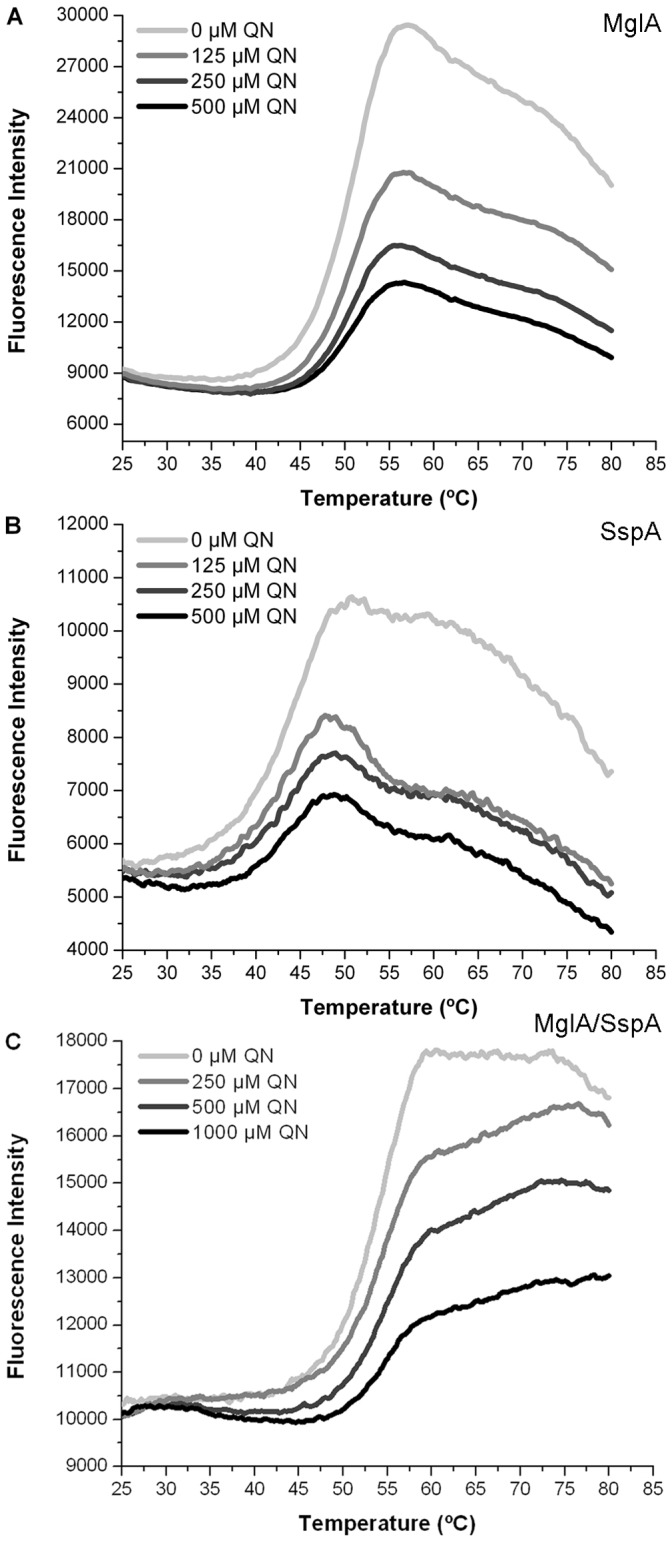
Quinacrine increases the thermal stability of MglA and SspA. Melting curves of purified (A) Ft-MglA (B) Ec-SspA and (C) Ft-MglA/Ft-SspA complex in absence or presence of increasing concentrations of quinacrine (125, 250 or 500 µM). Purified proteins (20 µM) were subjected to gradually increasing temperatures in the presence of the fluorophore SYPRO Orange. Fluorescence intensities were plotted against temperature and transition curves were fitted using the Boltzmann equation.

The midpoint transitions were determined to be 48.7±0.5°C for Ft-MglA, 42.4±0.3°C for Ec-SspA and 53.8±0.3°C for the Ft-MglA/Ft-SspA complex. The compounds that induced a shift in the midpoint transition temperature (expressed as *ΔTm*) by more than 2.0°C were considered hits. Using this technique, we identified compounds that interacted with Ft-MglA (13 chemicals) and Ec-SspA (10 chemicals) (for a complete list see Table 1). Proparacaine hydrochloride and retinoic acid were identified as the strongest thermo-stabilizing compounds for Ft-MglA, with a *ΔTm* of 24.5±4.4°C and 24.5±3.2°C, respectively. Additional compounds inducing significant stabilization of Ft-MglA included pamoic acid (20.4±2.9°C), flumequine (17.9±2.4°C), and ursolic acid (16.3±3.2°C). The strongest thermo-stabilizing compound for Ec-SspA was benzbromarone with a *ΔTm* of 30.0±1.6°C. Additional compounds inducing significant stabilization of Ec-SspA included benzethonium chloride (25.9±5.2°C), meclofenamic acid (22.9±4.1°C), and quinacrine dihydrochloride (15.2±1.7°C). Because the Ec-SspA and Ft-MglA proteins share 28% sequence identity, it was expected that some chemicals would bind to both proteins. Indeed, four chemicals (benzethonium chloride, proparacaine hydrochloride, retinoic acid and quinacrine dihydrochloride) all with strong thermal stabilization effect, overlapped between the two proteins. The results obtained were confirmed by analyzing the dose dependency, using increasing concentrations (up to 1 mM) of each chemical (data not shown). The compounds that overlapped between Ft-MglA and Ec-SspA were validated using the co-purified preparation of *F. tularensis* MglA and SspA proteins (Ft-MglA/Ft-SspA). The *Tm* for the Ft-MglA/Ft-SspA complex was established at 53.8°C. Quinacrine dihydrochloride (QN) had the major effect on the complex *Tm.* Furthermore, the *Tm* for the proteins (Ft-MglA, Ec-SspA, and Ft-MglA/Ft-SspA complex) increased proportionally to the concentration of quinacrine present (Figure 1).

**Table 1:** Effect of small molecules on the thermal stability of MglA or SspA and their effect on protein-protein interaction.

Chemical	ΔTm (°C)^1^		β-galactosidase Activity (%)^2^	*E. coli* MIC (mM)
**Ft-MglA**				
Arecoline hydrobromide	2.8±0.1		7.8±0.9	0.1
Carbamazepine	3.1±1.0		Not tested	Not tested
Diethylcarbamazine citrate	2.1±0.1		19.3±7.2	1.0
Flumequine	17.9±2.4		12.0±4.2	0.1
Haloperidol	4.6±1.7		18.0±3.2	0.1
Nabumetone	13.8±0.1		17.1±4.7	1.0
Pamoic acid	20.4±2.9		15.5±4.0	0.25
Theophylline monohydrate	2.3±0.1		6.5±2.1	0.1
Ursolic acid	16.3±3.2		16.5±1.2	1.0
**Ec-SspA**				
Benzbromarone	30.0±1.6		5.8±1.4	1.0
Captopril	9.2±1.9		16.2±3.0	1.0
Dipyrone	8.9±1.5		10.3±3.4	1.0
Harmalol hydrochloride	16.6±2.6		Not tested	Not tested
Meclofenamic acid	22.9±4.1		13.2±3.8	1.0
Tolfenamic acid	11.6±2.7		7.7±1.7	1.0
**MglA and SspA**	**Ft-MglA**	**Ec-SspA**		
Benzethonium chloride	8.5±1.7	25.9±5.2	18.9±4.8	0.05
Proparacaine hydrochloride	24.5±4.4	27.9±4.3	9.8±2.2	0.5
Quinacrine dihydrochloride	2.1±0.9	15.2±1.7	61.0±1.4	0.2
Retinoic acid	24.5±3.9	23.3±3.2	31.0±1.6	0.5

The thermal stabilization of each protein was evaluated using fluorometry with an average of 40 µM of ligand.

The chemicals were tested *in vivo* using the two-hybrid system at concentrations between 0.05–250 µM.

1 ΔTm was calculated as the difference in the transition temperature between the proteins in the absence (MglA = 48.7°C; SspA = 42.4°C) and presence of a given chemical. The results were averaged from duplicates.

2 β-galactosidase activity (expressed as arbitrary units) as a result of pBR-*mglA*-ω and pACTR-*sspA*-Zif interaction is expressed as the decrease in the activity in the presence of the chemicals, compared to the control without chemicals after 180 min. The assay was performed three times, each in duplicates.

### Small Molecules Modify the *F. tularensis* MglA/SspA Complex Interaction in a Two-hybrid System

The thermal stability observed from the binding of small molecules *in vitro* may result from interactions with the chemicals at locations that may or may not be functionally relevant. To identify the molecules that specifically modify the heterodimer interface, or “cleft” region, an *in vivo* assay using an *E. coli* two-hybrid system, modified from Charity *et al*. [8], was used. The plasmid pBRGP-ω was used to create the fusion of the *F. tularensis* SCHU S4 *mglA* gene, to the ω subunit of the RNAP. Plasmid pACTR-AP-Zif was used to fuse the *F. tularensis* SCHU S4 *sspA* gene, to the zinc finger DNA binding protein of the murine Zif268 domain. The reporter strain was constructed by deleting the *E. coli sspA* homolog in the FW102 strain [32] (to avoid unspecific interactions), which was then conjugated with the KDZif1ΔZ strain [8], to obtain the AW23 reporter strain (see materials and methods for more details).

**Figure 2:**
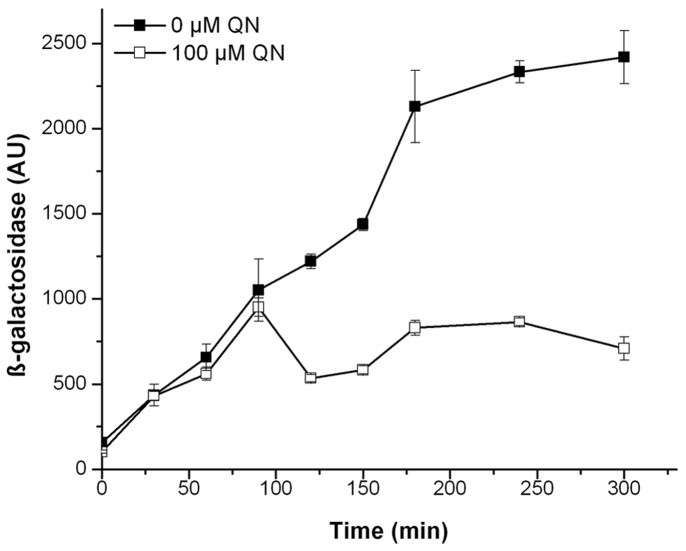
Quinacrine modifies the *F. tularensis* MglA and SspA interaction in a bacterial two-hybrid system. Transcription activation by the interaction between MglA and SspA from *F. tularensis* SCHU S4 decreases in the presence of quinacrine. The plasmid constructs pBR-*mglA*-ω and pACTR-*sspA*-Zif were transformed in the *E. coli* reporter strain AW23 (Δ*sspA*). Cells were grown in presence (open square) or absence (closed squares) of 100 µM quinacrine, and assayed for β-galactosidase activity (expressed in arbitrary units, AU). For ease of presentation the base level expression activity obtained with the empty plasmids (pACTR-AP-Zif and pBR-GP-ω; Fig. S1) was subtracted from those with pBR-*mglA*-ω and pACTR-*sspA*-Zif.

The interaction of MglA and SspA induced transcription of the β-galactosidase reporter gene, as previously described [8] (Figure 2). However, we observed significant levels of β -galactosidase activity in the empty plasmid controls (Figure S1). To ease the presentation of the results, the base level expression obtained with the empty plasmids (pACTR-AP-Zif and pBR-GP-ω) were subtracted from those with pBR-*mglA*-ω and pACTR-*sspA*-Zif. A decrease in β-galactosidase activity, upon the addition of ligands, would indicate that the compound modified the interaction between the MglA and SspA proteins. Seventeen compounds were individually tested at concentrations ranging from 50 nM to 250 µM, depending on the minimal inhibitory concentration (MIC) determined in *E. coli* (Table 1). Most of the compounds identified in the initial thermal screening of the individual proteins, failed to modify the interaction between Ft-MglA and Ft-SspA *in vivo*. Diethylcarbamazine citrate and haloperidol had a mild effect, and were found to decrease the β-galactosidase activity by 19.3±7.2% and 18.0±3.2%, respectively. Interestingly, three (benzethonium chloride, retinoic acid and quinacrine ) out of the four compounds that interacted with both Ft-MglA and Ec-SspA *in vitro*, also resulted in decreased interactions between Ft-MglA and Ft-SspA (β-galactosidase activity decreased by 18.9±4.8%, 31.0±1.6% and 61.0±1.4%, respectively). Since quinacrine was the small molecule with the strongest effect (61.0±1.4% decrease) on the interaction between Ft-MglA and Ft-SspA (Figure 2), further *in vitro* characterizations were performed using this chemical.

### Modifications in the Heterodimer Interface Decreased the Expression of Virulence Genes and Intramacrophage Survival of *F. tularensis*

**Figure 3:**
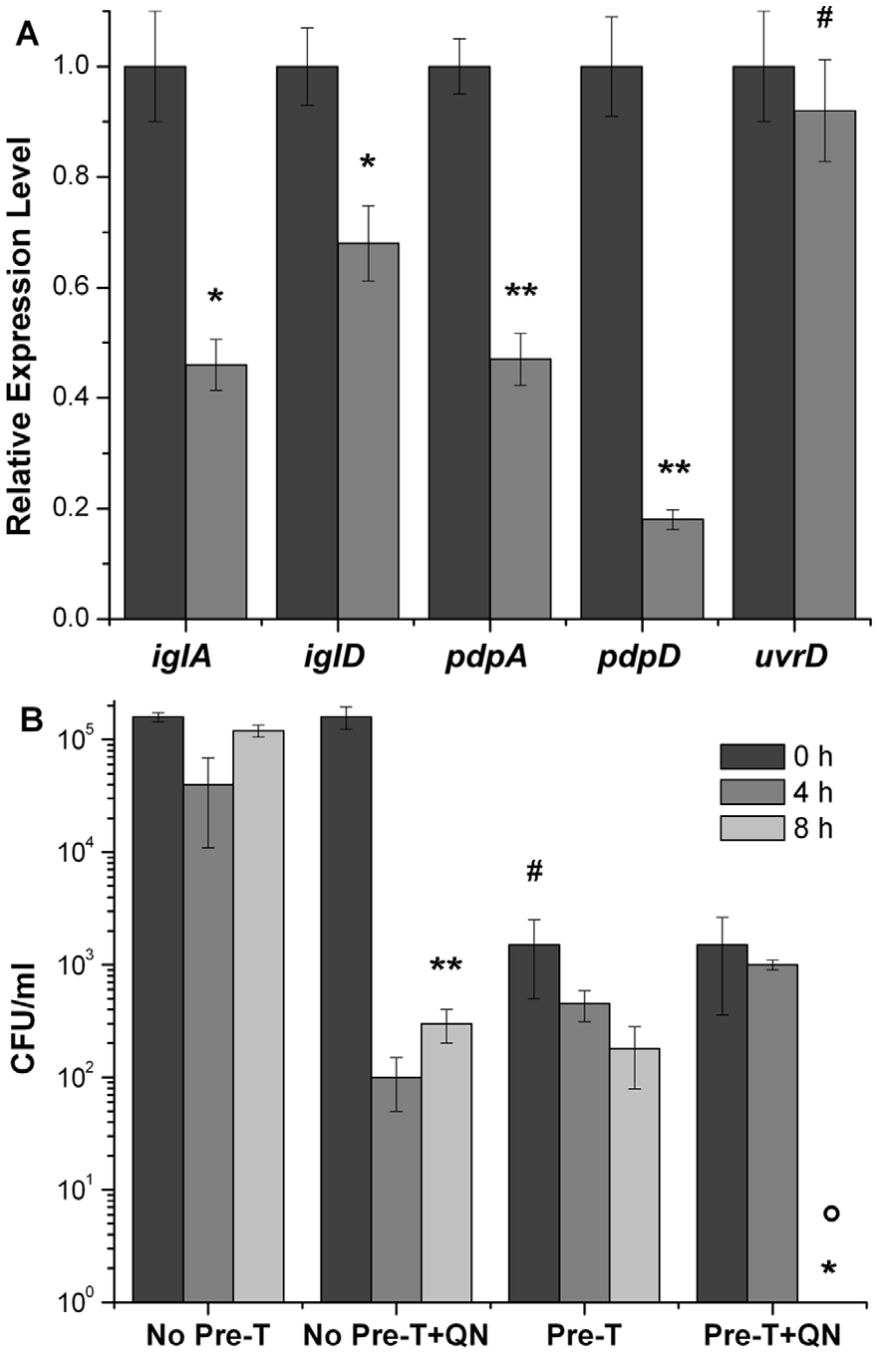
Quinacrine decreases the expression of FPI genes and *F. novicida* intramacrophage survival. (A) Transcript levels of *iglA, iglD, pdpA,* and *pdpD* in *F. novicida* grown in presence of quinacrine. *F. novicida* was grown to exponential phase in modified tryptic broth media, in presence (grey bars) or absence (dark grey bars) of 25 µM quinacrine. The amplification values obtained were corrected for those obtained using *rpsD* as an internal control. The values obtained with quinacrine are relative to the ones obtained without quinacrine.*P<0.01; **P<0.0005 indicates significant differences between relative expression of cells treated and not treated with quinacrine. ^#^ *P*>0.05 indicates no significant difference. (B) Survival of *F. novicida* within RAW264.7 cells. Macrophages were infected at a MOI of ∼ 15. Cells were lysed at 0 h (dark grey bars), 4 h (grey bars), and 8 h (light grey bars) post infection. Where indicated, 25 µM quinacrine (QN) was added to the macrophages after bacterial infection, and kept throughout the experiment. For the pre-treatment, macrophages were incubated with 25 µM quinacrine 30 min prior to infection. The assay was performed in duplicate, in three different experiments. * No colonies observed. ** indicates significant difference (*P*<0.0005) between the No Pre-T (time 8 h) and No Pre-T+QN (time 8 h) groups. ^#^ indicates significant difference (*P*<0.0001) between the No Pre-T (time 0 h) and Pre-T (time 0 h) groups. ° indicates significant difference (*P*<0.05) between the Pre-T (time 0 h and 8 h) and Pre-T+QN (time 8 h) groups.

A set of bioassays was performed to validate the biological relevance of quinacrine as a tool to modify the heterodimer interface of the MglA/SspA complex. *F. tularensis* subspecie *novicida* (*F. novicida*), was used as the model strain to establish a proof of principle. First, liquid cultures were used to determine the minimal concentration of quinacrine that would inhibit growth. *F. novicida* was able to grow with concentrations up to 200 µM, albeit at a slower rate (Figure S2). The doubling time of the microorganism was not affected with 25 µM, and was therefore the concentration used for subsequent studies. The modifications in the MglA/SspA complex, induced by quinacrine, were evaluated by measuring the expression of genes controlled by the MglA/SspA complex. RNA was isolated from exponential phase cells of *F. novicida,* grown in the absence and presence of 25 µM quinacrine. The expression of *iglA, iglD, pdpA,* and *pdpD* in the FPI, was measured by quantitative RT-PCR (qRT-PCR). The expression of *rspD* and *uvrD* were used as the internal control and negative control, respectively, since the latter is not under MglA/SspA regulation [12]. In presence of quinacrine, a decrease in the expression of the FPI genes *iglA, iglD, pdpA,* and *pdpD* was observed (2.1-; 1.5-; 2.2- and 5-fold, respectively) (Figure 3A).

During infection, MglA and SspA directly regulate the capability of *F. novicida* to activate the genes required to survive within the host phagocytic cells [33]. Increasing concentrations of quinacrine (up to 25 µM) were used to evaluate the ability of *F. novicida* to survive within a macrophage cell line (RAW 264.7). At these concentrations, macrophages were confirmed to remain viable by vital staining with trypan blue.

The intramacrophage survival of *F. novicida* was first evaluated by adding quinacrine to the macrophages 2 h post-infection (and kept throughout the experiment). At 25 µM quinacrine, a 2.6-log decrease in viability was observed after 8 h of treatment (Figure 3B). Pre-treating the macrophages with the same concentration of quinacrine, for 30 min prior to infection, also resulted in decreased in viable cell counts. This effect proved to be dose dependent, as shown by a corresponding decrease in *F. novicida* (CFU/ml) with increasing concentrations of quinacrine. Samples treated with 15 µM and 20 µM showed only a 1.2-log decrease in viability after 8 hours. At lower concentrations, bacterial counts were similar to those seen in the control group. The combination of treatments (pre and post-infection) resulted in the absence of viable *F. novicida* cells within macrophages, following 8 h of incubation (Figure 3B). These findings suggest that the presence of quinacrine impairs the ability of *F. novicida* to infect and survive within RAW 264.7 cells. It has been shown previously that *F. tularensis* mutants in *mglA* do not show decreased macrophage infection rates [12], [14], however, the role of SspA during infection has not been previously addressed. We hypothesized that the effect of quinacrine observed during infection in the pretreated group, could be the result of quinacrine binding to SspA and affecting its activity. We therefore tested the infection capabilities of a *F. novicida sspA* mutant strain and found a 2-log difference in infection when compared to the wild type strain (2×10^3^ CFU/ml and 1×10^5^ CFU/ml respectively). The observed decrease in the number of infecting cells is indicative of a role of SspA during infection. These results are in agreement with the data obtained from quinacrine pretreated macrophages.

Collectively, these results indicate that chemicals (such as quinacrine), that bind or modify the MglA/SspA heterodimer interface, may act to modulate further interactions with RNAP or other transcription factors (i.e., PmrA or FevR/PigR), affecting virulence gene expression *in vivo*.

### Quinacrine Induced Structural Modifications in the MglA/SspA Complex

**Figure 4:**
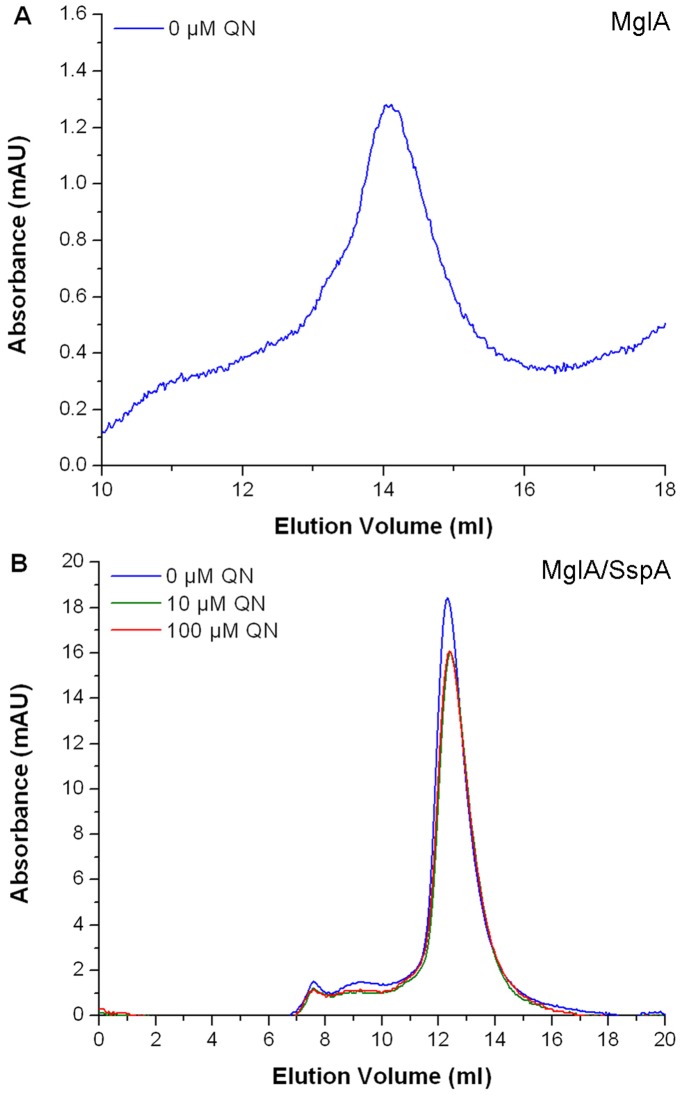
The oligomeric state of the MglA/SspA complex is not affected by quinacrine. Chromatograms of (A) Ft-MglA and (B) Ft-MglA/Ft-SspA complex in the absence (blue line) and presence of 10 µM (green line) and 100 µM (red line) quinacrine. 100 µl protein samples in 10 mM Tris (pH 8), 500 mM NaCl were injected onto a prepacked Superose 12 10/300 GL gel filtration column after incubation with quinacrine.

The decrease in β-galactosidase activity observed in the *in vivo* two-hybrid system upon the addition of quinacrine, may result from disruption of the heterodimer, or structural modifications in the dimer interface. To determine the effect of the small molecule on the heterodimer complex, the oligomeric state was determined by size exclusion chromatography, in both the presence and absence of quinacrine. Ft-MglA was used as a control, which elutes as a monomer under the same experimental conditions (Figure 4A). A similar chromatographic profile (of monomers and dimers) was observed in the presence and absence of quinacrine, with the Ft-MglA/Ft-SspA complex (Figure 4B). Contrary to previous observations in the two-hybrid system (Figure 2), these results indicate that the interaction with quinacrine does not affect the oligomeric state of the complex. It is postulated that the fusion domains can sterically hinder the interaction sites for a protein pair, resulting in the partial or complete inhibition of protein-protein interaction. However, while the unpredictable influence of the fusion domains in the two-hybrid system prohibits the determination or quantification of affinity between unrelated hybrid protein pairs, the observation of β-galactosidase activity is still a strong indication that the proteins do interact [34]. We believe that the *in vivo* results obtained from the two-hybrid system do not reflect the biological effect of quinacrine on the MglA/SspA complex in *Francisella*, and that the interaction with quinacrine results in structural modifications to the complex, as observed *in vitro*.

**Figure 5:**
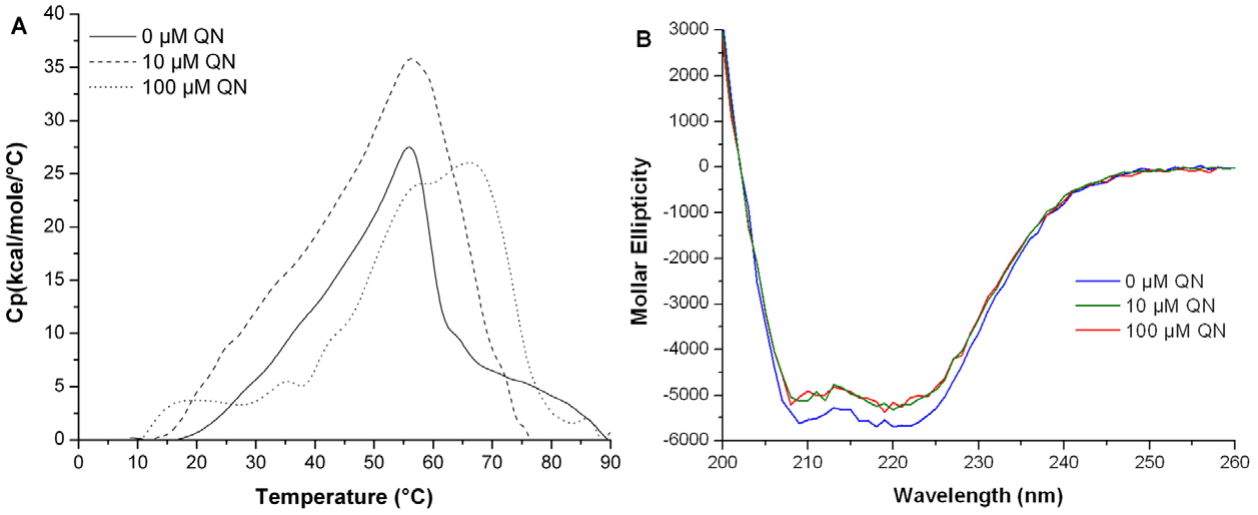
Quinacrine binds the MglA/SspA complex inducing structural modifications. (A) The effect of quinacrine on the thermal unfolding of the Ft-MglA/Ft-SspA complex was studied by DSC. (B) Circular Dichroism (CD) spectra of the Ft-MglA/Ft-SspA complex. The DSC experiments were performed in 10 mM phosphate (pH 7.9), 500 mM NaCl in the absence (solid line) or presence of 10 µM (dashed line) and 100 µM (dotted line) quinacrine. Protein concentration was 17 µM. The CD spectra were acquired at 10°C using 9.6 µM protein samples, with 0 µM (blue line), 10 µM (green line) and 100 µM (red line) quinacrine, in 10 mM Tris (pH 8.0), 150 mM NaCl.

To monitor the structural/conformational changes that the protein undergoes during quinacrine binding, circular dichroism (CD) measurements were performed. Negative bands of α-helical structures occur typically at 208 and 222 nm, and the bands observed for the Ft-MglA/Ft-SspA complex at ∼208 and ∼220 nm are indicative of a high helical content (Figure 5B). These results are in agreement with the predicted α-helix content of the cleft region in the modeled Ft-MglA/Ft-SspA structure. A secondary structure conformational change was induced by the addition of quinacrine (10 or 100 µM) (Figure 5B). The results of these analyses indicate that quinacrine binds to the *F. tularensis* MglA/SspA complex, affecting the α-helix component of the heterodimer. We hypothesize that the disruption of the complex observed in the two-hybrid system, was made possible by the combination of a weakened interaction between MglA and SspA (a consequence of the fusion domains) and the structural modifications induced by quinacrine.

Differential scanning calorimetry (DSC) was used to characterize the thermal unfolding properties of the Ft-MglA/Ft-SspA complex, both the presence and absence of quinacrine. The calorimetric scans (Figure 5) indicated that the proteins undergo a typical two-state endothermic unfolding transition in solution. The lower transition peak has a transition midpoint temperature (*Tm*) of 35.7°C, while the higher transition peak has a *Tm* of 54.2°C. The lower and higher transition temperatures were assigned *Tm_1_* and *Tm_2_*, respectively. In the presence of quinacrine, the Ft-MglA/Ft-SspA complex displayed a positive shift in the thermogram (Figure 5A). At 10 µM quinacrine, *Tm_1_* was 40.6°C (*ΔTm* 4.9°C) and *Tm_2_* was 57.7°C (*ΔTm* 3.5°C), whereas at 100 µM, *Tm_1_* was 59.7°C (*ΔTm* 24.0°C) and *Tm_2_* was 69.3°C (*ΔTm* 15.1°C). Collectively, these results indicate that quinacrine binds to the Ft-MglA/Ft-SspA complex, inducing modifications in the alpha helix component of the heterodimer, and increasing the thermal stability of the complex.

### Identification of Critical Residues Involved in Ft-MglA/Ft-SspA-quinacrine Interaction

**Figure 6:**
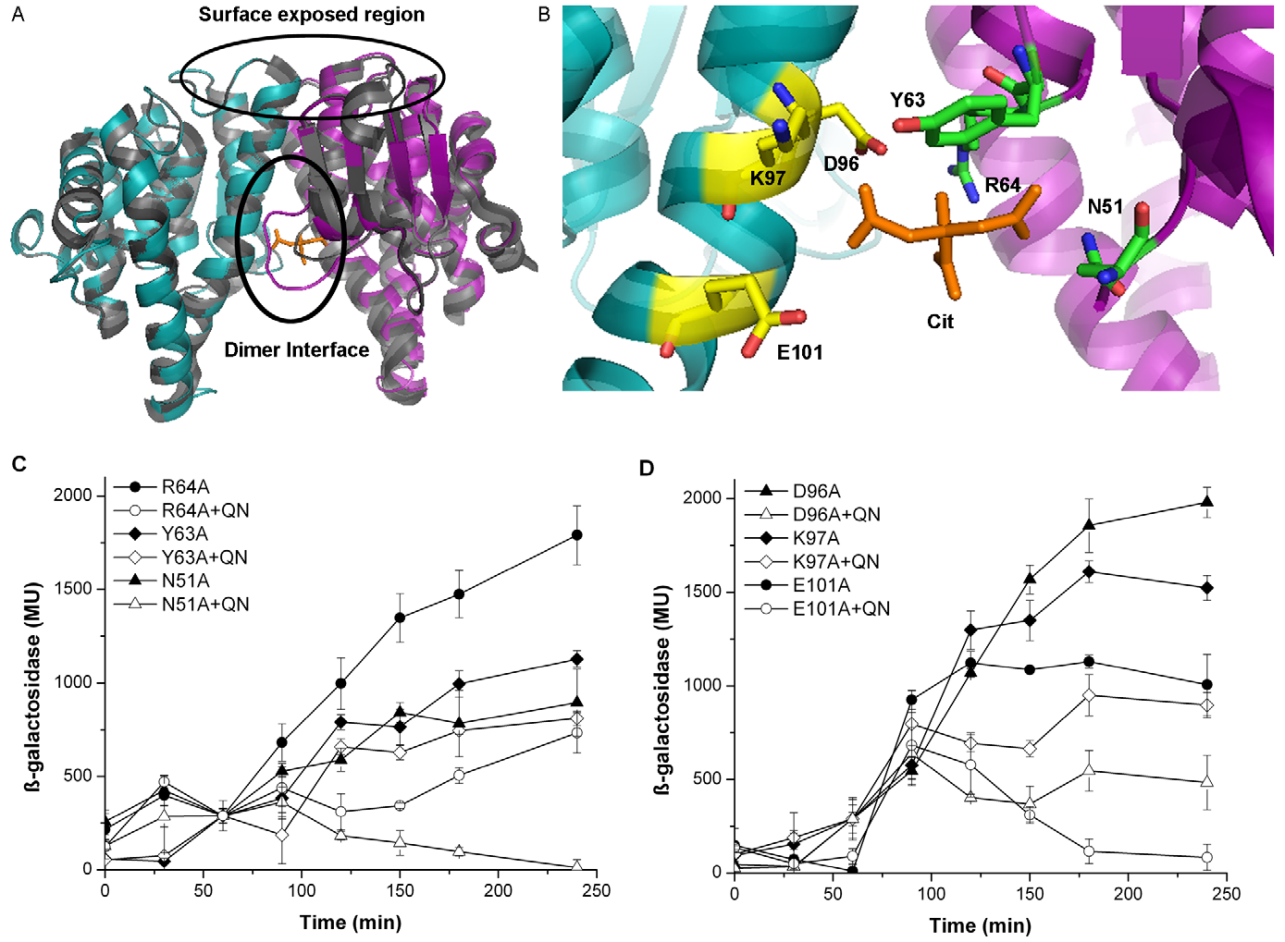
Structure model prediction of the *F. tularensis* MglA/SspA complex, and identification of critical amino acids involved in protein/ligand interaction. In A and B, *in silico* modeling was performed using SWISS-MODEL workspace. The structure of SspA from *Yersinia pestis* (PDB 1YY7) (grey) was used as the template to model Ft-MglA (magenta) and Ft-SspA (teal). The model was analyzed using PyMol. (A) Superposition of *Y. pestis* SspA dimer, Ft-MglA monomer, and Ft-SspA monomer. (B) Close-up view of the Ft-MglA/Ft-SspA interface residues (shown as sticks) from Ft-MglA (green) and Ft-SspA (yellow) around a 6 Å? distance from the citrate molecule (cit, orange) found in the *Y. pestis* PDB 1YY7. In C and D, β-galactosidase activity levels from cells carrying the pBR- *mglA*-ω and pACTR-*sspA*-Zif, with the shown mutations in either *mglA* or *sspA,* in the absence (closed symbols) or presence (open symbols) of 100 µM quinacrine (QN). (C) Residues mutated in Ft-MglA: N51 (triangle), Y63 (diamond), and R64 (circle). (D) Residues mutated in Ft-SspA: D96 (triangle), K97 (diamond), E101 (circle). β-galactosidase activity was determined as described in materials and methods.

To determine the specificity and location of quinacrine binding, a structural model of the *F. tularensis* MglA/SspA complex was constructed. The model (Figure 6A) was constructed based on the *Yersinia pestis* SspA structure (PDB 1yy7, [35]). *F. tularensis* SspA and MglA share a 28% and 21% identity with *Y. pestis* SspA, respectively. Functional studies with *E. coli* SspA (83% identical to *Y. pestis* SspA) have shown that the “surface exposed region” is involved in transcriptional activation, potentially through interactions with RNAP (Figure 6A) [35]. The cleft formed at the interface of the *Y. pestis* SspA monomers has a larger, open confirmation than that present in structural homologs Ure2p [36] or GST [36], [37]. We hypothesize that this region is the site modulated by the binding of small molecules (Figure 6A). Based on *in silico* analyses, we performed site-directed mutagenesis on various residues located within the interaction surface (cleft). A molecule of citric acid present in the *Y. pestis* SspA structure was used as a guide, and residues within 6 Å? were selected (Figure 6B). The cleft region is formed by residues N51, Y63 and R64 from Ft-MglA, and residues D96, K97 and E101 from Ft-SspA. These residues were mutated to alanine in the pBR-*mglA*-ω or the pACTR-*sspA*-Zif plasmids, and the effect of the mutations was then tested *in vivo,* in the absence of small molecules. As a control, a residue located outside of the heterodimer interface was included, K101 in Ft-MglA.

Interestingly, all mutations located in the heterodimer interface resulted in lower protein-protein interactions in the absence of small molecules, as evidenced by decreased levels of β-galactosidase activity (between 800 and 1900 arbitrary units, Figure 6C and 6D). In contrast, the Ft-MglA K101A mutant behaved as the wild type protein (2193±156 arbitrary units). The effect of quinacrine, in the MglA and SspA interaction, was then assessed in all mutants. As expected, the Ft-MglA K101A mutant showed a similar decrease (66.0%) in the β-galactosidase activity (801±73 arbitrary units) as previously observed with the wild type protein. The mutants Ft-MglA-N51A, Ft-MglA-R64A, Ft-SspA-K97A, Ft-SspA-D96A, and Ft-SspA-E101A showed different degrees of effects, ranging from 59.1% to 98.7% reduction in the β-galactosidase activity (Figure 6C and 6D). The mutations Ft-SspA-K97A and Ft-MglA-Y63A resulted in the smallest reduction in enzymatic activity in presence of quinacrine (41.1% and 28.2%, respectively). These results indicate that in the heterodimer interface, Ft-SspA-K97 and Ft-MglA-Y63 are the two significant residues involved in the interaction with quinacrine.

**Figure 7:**
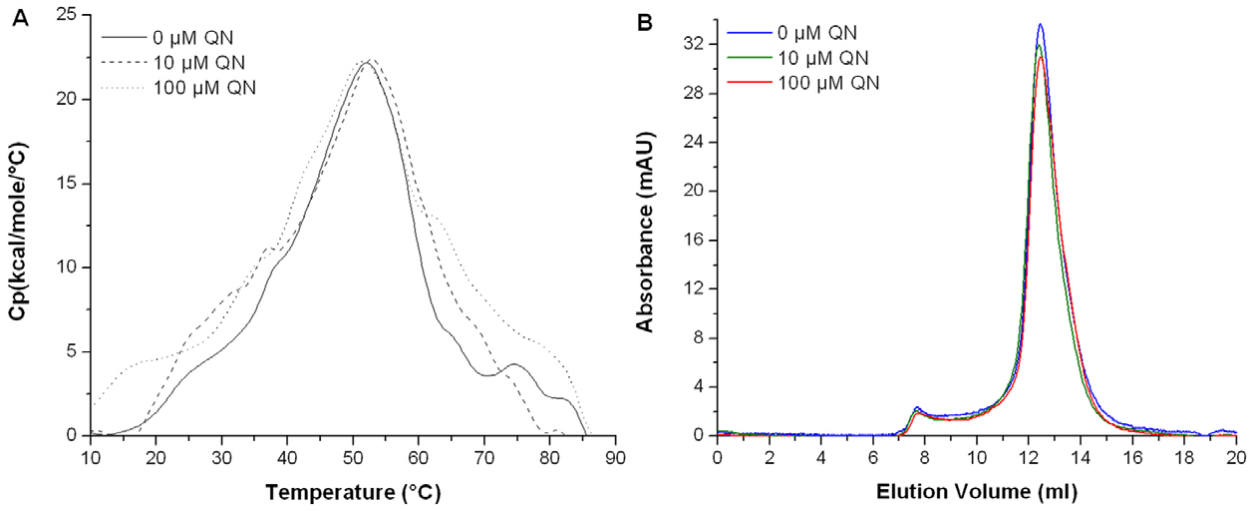
The thermal unfolding or oligomeric state of the Ft-MglAY63A/Ft-SspA complex is not affected by the presence of quinacrine. (A) DSC thermogram of Ft-MglAY63A/Ft-SspA complex. (B) Chromatogram of the Ft-MglAY63A/Ft-SspA complex in the absence (blue line) and presence of 10 µM (green line) and 100 µM (red line) quinacrine. The DSC experiment was performed in 10 mM phosphate (pH 7.9), 500 mM NaCl in the absence (solid line) or presence of 10 µM (dashed line) and 100 µM (dotted line) quinacrine (QN). Protein concentration was 22 µM.

The specific effect of quinacrine on the complex was tested on the Ft-MglAY63A/Ft-SspA mutant by DSC. The scans performed using the Ft-MglAY63A/Ft-SspA complex showed that unfolding occurred in two events, similar to the wild type complex. However, no significant changes in either *Tm_1_* or *Tm_2_* were detected after incubation with quinacrine (Figure 7). Resulting *Tm_1_* values were 32.7°C, 32.5°C and 32.7°C for 0, 10 µM and 100 µM quinacrine, respectively, while the *Tm_2_* values obtained were 51.8°C, 53.4°C and 52.1°C, for 0, 10 µM and 100 µM quinacrine, respectively. Additionally, the Ft-MglAY63A/Ft-SspA complex showed a profile of monomers and dimers similar to the Ft-MglA/Ft-SspA complex (Figure 7B), indicating that the oligomeric state of this complex is not affected by the mutation of Y63 in Ft-MglA, or addition of quinacrine. CD analysis was performed on MglA-Y63A to determine if conformational/structural changes had occurred as a result of the mutation. The wild type protein was used as a control. No differences were observed between MglA-Y63A and the wild type protein (data not shown).

## Discussion

The identification of small molecules that modulate protein-protein interactions is an expanding field with important therapeutic applications. We used a fluorescence-based thermal screening assay to uncover chemicals that bind the MglA/SspA heterodimer interface. Though small by today’s standards, the Prestwick chemical library was used to identify several compounds that bind Ft-MglA and Ec-SspA. Of the 1152 compounds present in the Prestwick library, 13 small molecules stabilized Ft-MglA *in vitro*. The number of positive hits obtained is similar to other transcription factors previously screened in our laboratory [31], [38], [39]. A secondary confirmation assay was designed to link the thermal screening results to the biological function of the target protein. Since MglA and SspA form a heterodimer to bind the RNA polymerase [8], we used a bacterial two hybrid system as a secondary assay. The rationale was to determine which of the 13 small molecules modulate MglA and SspA interactions. Interestingly, we found that only one compound, quinacrine, decreased the interaction of Ft-MglA and Ft-SspA by more than 50%. The other 12 compounds examined in the two hybrid system were determined to be biologically insignificant, as no major reduction in MglA/SspA interaction was observed. The thermal stabilization effect observed with these compounds was most likely due to unspecific binding at locations other than the heterodimer interface. Another possibility, however, is a deficiency in the uptake of those chemicals by the *E. coli* reporter strain.

The decrease in MglA/SspA interactions observed in the two hybrid system, in the presence of quinacrine, was correlated with impaired intramacrophage survival of *F. novicida* and decreased expression of the FPI genes. An interesting observation was that *F. novicida* could tolerate rather high concentrations of quinacrine (up to 100 uM) when cultured in liquid media (albeit with reduced expression of FPI genes), while 25 uM was sufficient to decrease *F. novicida* macrophage infection and intramacrophage survival. These results confirmed the specificity of quinacrine for the MglA/SspA heterodimer interface, and the importance of the MglA/SspA complex in the modulation of expression of pathogenicity genes during host invasion. A similar phenomenon was reported for inhibitors of the QseC membrane histidine kinase in *Salmonella typhimurium* and *F. tularensis* [40]. The addition of LED209 did not influence the growth of *Salmonella typhimurium in vitro,* while it diminished the expression of virulence genes by 3-fold. This small decrease in the expression of the *sifA* pathogenicity gene, which is required for the establishment of *S. typhimurium* in the host, was sufficient to reduce bacterial counts in the liver and spleen of infected animals by 10-fold. Similarly, LED209 reduced the expression of FPI genes *iglC* and *pdpA* in *F. tularensis* by 3-fold *in vitro,* while clearing the infection *in vivo* [40].

Quinacrine has been used as an antimalarial agent in humans [41], as an *in vitro* anti-prion agent [42], and as a neutralizing agent for *Bacillus anthracis* [43]. While most research in these systems is oriented to isolate compounds with better affinities and lower host toxicity, scarce information is available regarding the specific residues involved in such interactions. Quinacrine disrupts protein-protein interactions in the anti-apoptotic member Bcl-xl [44], by specifically binding in the hydrophobic grove, competing with the regulatory peptide BH3 [44], [45]. As a desired result, apoptosis was induced in cancer cells. To determine the specific residues involved in the binding to quinacrine, a model of the tertiary structure of the MglA/SspA complex was built. Subsequently, site directed mutagenesis was performed in the heterodimer interface, where several residues were identified that affected the binding of quinacrine. Mutations to residues Y63 in Ft-MglA, and K97 in Ft-SspA had the greatest effect on the binding of quinacrine, as determined in the two-hybrid system. These results suggest the importance of these residues during interactions of the complex with quinacrine. However, the putative use of quinacrine as a therapeutic agent for the treatment of tularemia disease would require further chemical engineering to improve the affinity for the Ft-MglA/Ft-SspA complex. Recently, Mays *et al*. [46] have used chemical synthesis to improve the antiprion activity of quinacrine derivatives that bind PrP with higher affinity. Based on the results obtained here, we propose the use of quinacrine as a chemical probe to uncover biologically relevant molecules that may modulate Ft-MglA/Ft-SspA activity, by binding in the heterodimer interface.

## Experimental Procedures

### Bacterial Strains and Growth Conditions

The bacterial strains and plasmids used in this study are listed in Table 2. *F. novicida* U112 and Fn-Δ*sspA* were routinely cultured at 37°C with aeration, in modified tryptic soy broth (TSB) (Difco Laboratories, Detroit, MI) containing 135 µg/ml ferric pyrophosphate and 0.1% cysteine hydrochloride. For CFU enumeration, cysteine heart agar medium (Difco) supplemented with 1% hemoglobin solution (BD Diagnostics, Sparks, MD) (chocolate II agar plates-CHOC II) was used. *E. coli* strains were grown at 37°C under aerobic conditions in Luria-Bertani mediun (LB) (Difco) or on LB agar plates. *E. coli* strains DH5α (Invitrogen, Carlslab, CA), XL1-Blue and JM109 (Stratagene, La Jolla, CA) were used to propagate the plasmids for protein purification, point mutations, and two-hybrid systems, respectively. *E. coli* strain BL21-Star(DE3) (Invitrogen) was used to expressed individual Ft-MglA and Ft-SspA His-tagged fusion proteins for purification, and BL21-Rosetta(DE3) (Novagen, Gibbstown, NJ) was used when Ft-MglA and Ft-SspA were co-expressed. The strain to purify *E. coli* SspA (SspA-ASKA) was obtained from the ASKA library [47]. When required, the medium was supplemented with ampicillin (100 µg/ml), tetracycline (10 µg/ml), kanamycin (50 µg/ml), or streptomycin (50 µg/ml). All antibiotics and chemicals were purchased from Sigma (St. Louis, MO).

**Table 2:** Bacterial strains, and plasmids used in this study.

Strain, or plasmid	Genotype, or description	Reference, or source
**Strains**		
*F. novicida*	*F. tularensis* subsp. *novicida* U112 strain	[33]
Fn-Δ*sspA*	*F. novicida* Δ*sspA*::KAN-2; Km^r^	[52]
* E. coli*		
DH5α	F^–^ Φ80*lac*ZΔM15 Δ(*lac*ZYA-*arg*F) U169 *rec*A1 *end*A1 *hsd*R17 (r_K_^–^, m_K_^+^) *pho*A *sup*E44 λ– *thi*-1 *gyr*A96 *rel*A1	Invitrogen
BL21-Star(DE3)	F^–^ *omp*T *hsd*S_B_(r_B_^–^, m_B_^–^) *gal dcm rne131* (DE3); Str^r^	Invitrogen
BL21-Rosetta(DE3)	F^–^ *ompT hsdS*_B_(r_B_^–^ m_B_^–^) *gal dcm* (DE3) pRARE	Novagen
Ft-MglA	BL21-Star(DE3) carrying p15TV-*mglA*; Amp^r^	This work
Ft-MglA/SspA	BL21-Rosetta(DE3) carrying p15TV-*mglAand* pCDF-*sspA*; Amp^r^, Str^r^	This work
MglAY63A/SspA	BL21-Rosetta(DE3) carrying compatible vectors p15TV-*mglA*Y63A and pCDF-*sspA*; Amp^r^, Str^r^	This work
Ec-SspA	*E. coli* K-12, strain W3110 carrying pCA24N-*sspA*; Cm^r^	[47]
XL1-Blue	*recA1 endA1 gyrA96 thi-1 hsdR17 supE44 relA1 lac* [F *proAB lacI* ^q^ *Z*Δ*M15* Tn*10* (Tet^r^)]	Stratagene
JM109	e14^–^(McrA^–^) *recA1 endA1 gyrA96 thi-1 hsdR17* (*r*_K_^–^ *m*_K_^+^) *supE44 relA1* Δ(*lac-proAB*) [F *traD36 proAB lacI* ^q^ *Z*Δ*M15*]	Stratagene
FW102	[F^−/^ *araD(gpt-lac)5* (*rpsl*::Str )]; Str^r^	[32]
AW18	[F^−/^ *araD(gpt-lac)5* (*rpsl*::Str ) *ΔsspA*]; Str^r^	This work
KDZifΔZ	[F’*lacproA+,B+(lacI^q^ lacPL8)/araD(gpt-lac)5* (D*spoS3*::cat )]; Km^r^	[8]
AW23	AW18 conjugated with KDZifΔZ; Str^r^, Km^r^	This work
AW23-1	AW23 carrying pBR-*mglA*-ω and pACTR-*sspA*-Zif	This work
AW23-2	AW23 carrying pBR-GP-ω and pACTR-AP-Zif	This work
AW23-3	AW23 carrying pBR-*mglA*-ω and pACTR-AP-Zif	This work
AW23-4	AW23 carrying pBR-GP-ω and pACTR-*sspA*-Zif	This work
AW23-5	AW23 carrying pBR-*mglA*N51A-ω and pACTR-*sspA*-Zif	This work
AW23-6	AW23 carrying pBR-*mglA*Y63A-ω and pACTR-*sspA*-Zif	This work
AW23-7	AW23 carrying pBR-*mglA*R64A-ω and pACTR-*sspA*-Zif	This work
AW23-8	AW23 carrying pBR-*mglA*-K101A-ω and pACTR-*sspA*-Zif	This work
AW23-9	AW23 carrying pBR-*mglA*-ω and pACTR-*sspA*D96A-Zif	This work
AW23-10	AW23 carrying pBR-*mglA*-ω and pACTR-*sspA*K97A-Zif	This work
AW23-11	AW23 carrying pBR-*mglA*-ω and pACTR-*sspA*E101A-Zif	This work
**Plasmids**		
p15TV-L	Expression vector for protein purification, GenBank accession: EF456736; Amp^r^	Structural GenomicConsortium, Toronto
p15TV-*mglA*	p15TV-L carrying *mglA* gene from *F. tularensis* SCHU S4	This work
p15TV-*mglA*Y63A	p15TV-*mglA* with MglA Y63 **→** A	This work
pCDF-1b	Expression vector for protein purification; Str^r^	Novagen
pCDF-*sspA*	pCDF-1b carrying *sspA* gene from *F. tularensis* SCHU S4	This work
pKD46	λ red expression vector, thermosensitive-30°C; Amp^r^	[48]
pKD4	Plasmid used as the source of the kanamycin resistance marker; Km^r^	[48]
pCP20	Helper plasmid, FLP recombinase, thermosensitive-30°C; Amp^r^, Cm^r^	[48]
pBRGP-ω	Plasmid used to create fusions to the N-terminus of the ω subunit of *E. coli* RNA polymerase; Car^r^, Amp^r^	[8]
pBR-*mglA*-ω	pBRGP-ω carrying *mglA* gene from *F. tularensis* SCHU S4 at *Nde*I and *Not*I sites	This work
pBR-*mglA*N51A-ω	pBR-*mglA*-ω with MglA N51 **→** A	This work
pBR-*mglA*Y63A-ω	pBR-*mglA*-ω with MglA Y63 **→** A	This work
pBR-*mglA*R64A-ω	pBR-*mglA*-ω with MglA R64 **→** A	This work
pBR-*mglA*K101A-ω	pBR-*mglA*-ω with MglA K101 **→** A	This work
pACTR-AP-Zif	Plasmid used to create fusions to the N-terminus of the Zif protein; Tet^r^	[8]
pACTR-*sspA*-Zif	pACTR-AP-Zif carrying *sspA* gene from *F. tularensis* SCHU S4 at *Nde*I and *Not*I sites	This work
pACTR-*sspA*D96A-Zif	pACTR-*sspA*-Zif with SspA D96 **→** A	This work
pACTR-*sspA*K97A-Zif	pACTR-*sspA*-Zif with SspA K97 **→** A	This work
pACTR-*sspA*E101A-Zif	pACTR-*sspA*-Zif with SspA E101 **→** A	This work

Str^r^, Km^r^, Amp^r^, Cm^r^, and Tet^r^ indicate resistant to streptomycin, kanamycin, ampicillin, chloramphenicol, and tetracycline, respectively.

Y, K, D, R, E, N, and A are amino acids tyrosine, lysine, aspartic acid, arginine, glutamic acid, asparagine, and alanine, respectively.

*E. coli* strain AW23 was used as the reporter strain for the bacterial two-hybrid experiments. The strain was constructed as follows. An *E. coli sspA* knockout mutant (AW18) was constructed in strain FW102 [32] using the protocol described by Datsenko and Wanner [48]. The reporter strain KDZif1ΔZ harbors an F’ episome, containing the *lac* promoter derivative p*lac*Zif1-61, driving expression of a linked *lacZ* reporter gene [8]. KDZif1ΔZ was used as the donor for conjugation (previously described in [49]) of the recombinant F’ plasmid, into the strain AW18, resulting in the reporter strain AW23.

### DNA Manipulations and Gene Cloning

Standard methods were used for chromosomal DNA isolation, restriction enzyme digestion, agarose gel electrophoresis, site-directed mutagenesis, ligation, and transformation [50]. Plasmids were isolated using spin miniprep kits (Qiagen, Valencia, CA) and PCR products were purified using QIAquick purification kits (Qiagen). All the primers used are described in Table 3.

**Table 3:** Oligonucleotides used in this study.

Primer	Sequence (5′→3′)
**Protein Purification**	
MglA pETV-Fw	ttgtatttccagggcatgcttttatacacaaaaaaagatgatatctatagc
MglA pETV-Rv	caagcttcgtcatcattaagctccttttgctttgatag
SspA pCDFBamHI-Fw	ccggatcccatgatgaaagttacattatatacaacg^a^
SsaA pCDF-NotI-Rv	gcggccgcattatctatgagttcttagagttttgag^a^
**Two-hybrid system cloning**	
MglA NdeI-Fw	ggaattccatatgatgcttttatacacaaaaaaagatg^a^
MglA NotI-Rv	tatatgcggccgcagctccttttgctttgatag^a^
SspA NdeI-Fw	ggaattccatatgatgatgatgaaagttacattatatacaacg^a^
SspA NotI-Rv	tatatgcggccgctctatgagttcttagagttttg^a^
**qRT-PCR**	
iglA-Fw	aatgtccttagcaaacgatgc
iglA-Rv	cttttgattttgaggcacca
iglD-Fw	gccctattagattccgcaaa
iglD-Rv	gagggcgattagtaccagaaa
pdpA-Fw	caacccgttttatagccattg
pdpA-Rv	ggatggtttgtgcttagtcca
pdpD-Fw	atctgccccaacactaccag
pdpD-Rv	gctcagcaggattttgatttg
rpsD-Fw	tgtcgaagctagcagaagaaa
rpsD-Rv	gccagcttttacttgagcaga
uvrD-Fw	accgccataaatccgatatg
uvrD-Rv	cagcagctgaagatggtgaa
**Chromosomal Genes Disruption**	
* sspA*-Fw	ttaactccggcccagacgcatttcacgttctgcttcagttaaagaagcaa**gtgtaggctggagctgcttcg** b
* sspA*-Rv	atggctgtcgctgccaacaaacgttcggtaatgacgctgttttccggtcc**catatgaatatcctccttag** b

a Underlines indicate the restriction sites.

b Bold indicates the priming site.

For protein expression and purification, the *mglA* and *sspA* genes were amplified from *F. tularensis* SCHU S4 chromosomal DNA (provided by Dr. Tara Wherly, Rocky Mountain Labs/NIAID/NIH, Hamilton, MT) by PCR. The plasmid p15TV-L was employed to clone *mglA*, whereas pCDF-1b was used as the vector to clone *sspA*.

For the bacterial two-hybrid system, the *F. tularensis* SCHU S4 *mglA* gene was amplified by PCR, and fused to the ω subunit of the RNAP by cloning in the *Nde*I and *Not*I sites of the pBRGP-ω plasmid, to obtain pBR-*mglA*-ω [8]. In addition, the *sspA* gene from *F. tularensis* was cloned in the plasmid pACTR-AP-Zif, at the *Nde*I and *Not*I sites, and fused to the zinc finger DNA binding domain of the murine Zif268 protein, to obtain pACTR-*sspA*-Zif. Recombinant clones were obtained in *E. coli* JM109, confirmed by sequencing, and transformed into the reporter strain (AW23) by natural competence, to obtain the AW23-1 strain.

Site-directed mutagenesis was performed using the QuikChange Site-directed Mutagenesis kit (Stratagene) according to the manufacturer’s protocol. Plasmids pBRGP-*mglA-*ω, pACTR-*sspA*-Zif, and p15TV-*mglA* were used as the templates. All mutated amino acids were changed to alanine. Mutations were verified by DNA sequencing.

### Protein Purification

Protein purification was performed as previously described [38]. Briefly, the His-tagged fusion proteins (p15TV-*mglA,* pCA24N-*sspA*) were overexpressed in *E. coli* BL21-Star(DE3) cells (Invitrogen) and pCDF-*sspA* and p15TV-*mglA*/pCDF-*sspA* were overexpressed in *E. coli* BL21-Rosetta(DE3) (Novagen). The cells were grown in LB broth at 30°C to an OD_600_ of ∼ 0.6 and expression was induced with 0.5 mM isopropyl 1-thio-β-D-galactopyranoside (IPTG). After induction, the cells were incubated at 17°C for 16 h. The cells were harvested, resuspended in binding buffer (500 mM NaCl, 5% glycerol, 50 mM Tris, (pH 8.0), 5 mM imidazole), and stored at −80°C. The thawed cells were lysed and passed through the french press after the addition of 1 mM phenylmethylsulfonyl fluoride. The lysates were clarified by centrifugation (30 min at 17,000×*g*) and applied to a metal chelate affinity column charged with Ni^2+^. The column was washed (in binding buffer with 15 mM imidazole) and the proteins were eluted from the column in elution buffer (binding buffer with 250 mM imidazole). The purified proteins were dialyzed against 10 mM Tris (pH 8.0), 500 mM NaCl, 2.5% glycerol and store at −80°C. The identity of the purified proteins was confirmed by Mass Spectrometry (as a service in the Interdisciplinary Center for Biotechnology Research, University of Florida) on protein bands isolated from SDS-PAGE gels.

### Small Molecules Library Screening

Purified Ft-MglA, Ec-SspA and the Ft-MglA/Ft-SspA complex were screened against the Prestwick chemical library (Prestwick Chemical, France) at a final concentration of 20 µg/mL, using differential scanning fluorometry as previously described [30], [31]. This library of 1152 compounds offers a good diversity of chemical scaffolds, which are off patent and safe for use in humans. 25 µl of protein sample (20 µM), containing the chemical compound, was placed (in duplicate) into 96-well plates (Bio-Rad, Hercules, CA) and heated from 25 to 80°C at the rate of 1°C min^−1^. The unfolding of the proteins was monitored by the increase in fluorescence of the fluorophore SYPRO Orange (Invitrogen). Fluorescence intensities were plotted against temperature for each sample well, and transition curves were fitted with the Boltzmann equation using Origin 8 software (Northampton, MA). The midpoint of each transition curve was calculated and compared to the midpoint calculated for the reference sample. If the difference between them was greater than 2.0°C, the corresponding compound was considered to be a “hit” and the experiment was repeated to confirm the effect in a dose-dependent manner.

### β-galactosidase Assays

*E. coli* cells were grown at 37°C in LB broth with aeration, in the presence and absence of the compounds. Gene expression of the fusion proteins, in the AW23-1 strain, was induced by the addition of 0.5 mM lactose, at an OD_600_ of 0.2. Culture samples were taken every 30 min, permeabilized with 0.15% sodium dodecyl sulfate (SDS) and 1.5% chloroform in Z-buffer (60 mM Na_2_HPO_4_⋅7H_2_O, 40 mM NaH_2_PO_4_⋅H_2_O, 10 mM KCl, 1 mM MgSO_4_, 50 mM β-mercapthoethanol), and assayed for β-galactosidase activity by following the catalytic hydrolysis of the chlorophenol red-β-D-galactopyranoside (CRPG) substrate. Absorbance at 570 nm was read continuously using a Synergy HT 96-well plate reader (Bio-Tek Instruments Inc., Winooski, VT). β-galactosidase activity is expressed in arbitrary units (AU) [51]. Assays were performed in duplicates at least three times. The basal AU was determined from assays performed with the empty plasmids (pACTR-AP-Zif and pBR-GP-ω). For ease of presentation, the basal AU has already been subtracted from pBR-*mglA*-ω and pACTR-*sspA*-Zif.

### RNA Isolation and Quantitative RT-PCR

*F. novicida* was cultured in modified TSB broth (Difco) in the presence (25 µM) and absence of quinacrine. The cells were collected by centrifugation during exponential phase. Total RNA was isolated with a RiboPure™ Bacteria kit (Ambion, Austin, TX) in accordance with the manufacturer’s protocol. cDNAs were synthesized with the Superscript™ first-strand synthesis kit (Invitrogen) in accordance with the manufacturer’s instructions, and stored at −80°C prior to use. Quantitative RT-PCR was carried out in the iCycler, IQ device (Bio-Rad) using Platinum® SYBR® Green qPCR SuperMix for iCycler (Invitrogen) in accordance with the manufacturer’s recommended protocol. The genes measured were *iglA*, *iglD*, *pdpA,* and *pdpD* encoded in the FPI, and *uvrD* that is not regulated by the MglA/SspA transcriptional regulators, as a control (sequences available in Table 3). The *rpsD* gene was used as the internal control.

### Intramacrophage Survival Assay

*F. novicida* U112 was grown overnight on CHOC II plates, then resuspended in PBS to a concentration of ∼10^9^ CFU/ml (OD_600_ = 1). RAW 264.7 macrophages were seeded into 24-well culture plates at 2.5×10^5^ cells/well in RPMI-1640 media (Sigma) supplemented with 10% fetal bovine serum and 1% penicillin/streptomycin solution. The plates were incubated at 37°C in a humidified incubator containing 5% CO_2_.

Monolayers were infected with *F. novicida* at a multiplicity of infection (MOI) of 15∶1. The plates were centrifuged at 800×*g* for 15 min to facilitate infection, and incubated at 37°C. After two hours of infection, monolayers were washed and treated with fresh media containing gentamicin (50 µg/ml) for 30 min to remove extracellular bacteria. Cells were washed twice with 1X phosphate-buffered saline (PBS), and replenished with fresh media containing gentamicin (10 µg/ml) and increasing concentrations (0 to 100 µM) of quinacrine (three wells per group). Control wells (not infected) were treated with gentamycin or gentamycin and quinacrine as required. Where indicated, macrophages were pre-treated with 25 µM quinacrine for 30 min prior to infection, or after infection. The cells were lysed at different time points with 0.01% sodium deoxycholate in 1X PBS. The lysates were serially diluted, then plated onto CHOC II plates for determination of viable cell counts. Plates were incubated at 37°C for 24 h prior to counting colonies.

### Structure Modeling and Analysis

*In silico* modeling was performed using SWISS-MODEL workspace (http://swissmodel.expasy.org/workspace/). The structure of SspA from *Yersinia pestis* (PDB 1YY7) [35] was used as the template to model MglA and SspA from *F. tularensis* SCHU S4. The predicted structure was analyzed using PyMol (http://www.pymol.org/).

### Size Exclusion Chromatography

Size exclusion chromatography was performed using 100 µl protein samples. Aliquots contained 20 µM Ft-MglA, 24 µM Ft-MglA/Ft-SspA or 30 µM Ft-MglAY63A/Ft-SspA, and where indicated, 10 or 100 µM quinacrine, prepared in 10 mM Tris (pH 8), 500 mM NaCl. Following 30 min of incubation on ice, samples were injected onto a prepacked Superose 12 10/300 GL (GE Healthcare, Sweden) gel filtration column, connected to a LCC-501 plus (Pharmacia Biotech Inc., Piscataway, NJ) equilibrated with 10 mM Tris (pH 8.0), 500 mM NaCl. Filtration was carried out at 4°C, using a flow rate of 0.5 ml/min. The eluted proteins were monitored continuously for absorbance at 280 nm using a UV-M II monitor (Pharmacia Biotech Inc.). Blue dextran 2000 was used to determine the void volume of the column. A mixture of protein molecular weight standards, containing IgG (150 kDa), BSA (66 kDa), Albumin (45 kDa), Trypsinogen (24 kDa), Cytochrome C (12.4 kDa), and Vitamin B12 (1.36 kDa) was also applied to the column under similar conditions. The elution volume and molecular mass of each protein standard was then used to generate a standard curve from which the molecular weight of eluted proteins was determined.

### Differential Scanning Calorimetry (DSC)

DSC measurements were carried out using a MicroCal VP-DSC differential scanning microcalorimeter (MicroCal LLC, Northampton, MA). Protein samples were extensively dialyzed against a buffer with 10 mM phosphate (pH 7.9), 500 mM NaCl. Quinacrine solutions were prepared in dialysis buffer. Prior to loading, all samples were degassed for 30 min at 4°C using a ThermoVac degassing station (MicroCal). Fresh dialysis buffer was used in the reference cell. Samples treated with quinacrine (10 or 100 µM) were incubated at 4°C for 30 min, prior to DSC analysis. Quinacrine was also added to the reference buffer at equal concentrations. A scan rate of 45°C h^−1^ was used for all experiments, with constant pressure (25 psi) applied to both cells throughout each run. Buffer scans, recorded in the presence or absence of quinacrine, were subtracted from the corresponding thermograms before analysis. Data was analyzed using the Origin software supplied by the manufacturer (MicroCal). Curves were fit to the data using the non-two-state transition model.

### Circular Dichroism (CD)

UV CD spectra (180–300 nm) of the Ft-MglA/Ft-SspA and Ft-MglAY63A/Ft-SspA complexes were recorded on a Circular Dichroism Spectrometer Model 400 (Biomedical, Inc. Lakewood, NJ) at 10°C, in a buffer consisting of 10 mM Tris (pH 8.0), 150 mM NaCl. Samples treated with quinacrine (10 or 100 µM) were incubated at 4°C for 30 min, prior to analysis. A 1 mm path length cell was used for the measurement, and parameters were set as follows: bandwidth, 1 nm; step resolution, 1 nm; scan speed, 50 nm min^−1^; response time, 1 sec. Each spectrum was obtained from an average of 10 scans. Spectra were recorded every 9 min after equilibration. All spectra were corrected by subtracting the control scan with buffer (with or without corresponding quinacrine concentrations). The final spectra was expressed in molar ellipticity (ME) using the formula ME = θ/10nCl, where θ is the signal acquired, n is the number of residues, C is the molar concentration of protein, and l is the pathlength of the cuvette.

### Statistical Analysis

Statistical analysis for significant differences was performed according to the *t*-test for unpaired data, or by the nonparametric one way ANOVA. Differences with *P*<0.05 and lower were considered significant. Data was analyzed by GraphPad Prism (GraphPad Software, San Diego, USA).

## Supporting Information

Figure S1 **Transcription activation by the interaction between Ft-MglA and Ft-SspA fusion proteins.** Different combinations of empty vector and fused proteins were transformed in the *E. coli* reporter strain AW23 (Δ*sspA*) and β-galactosidase activity was determined. The plasmid constructs tested were pBR-GP-ω/pACTR-AP-Zif (open diamond, W+Zif), pBR-GP-ω/pACTR-*sspA*-Zif (blue triangle, W+SspA-Zif), pBR-*mglA*-ω/pACTR-AP-Zif (orange diamond, MglA-W+Zif), and pBR-*mglA*-ω/pACTR-*sspA*-Zif (black diamond).
(TIF)

Figure S2 **Growth of** ***F. novicida*** **in the presence of quinacrine**. The bacterial cells were inoculated in modified TSB containing increasing concentrations of quinacrine (10 to 200 µM). The OD_600_ was recorded at different time points. The quinacrine concentrations tested were 0 µM (brown circle), 10 µM (right purple triangle), 25 µM (left grey triangle), 50 µM (green diamond), 75 µM (down pink triangle), 100 µM (up blue triangle), 150 µM (red circle) and 200 µM (orange square).
(TIF)
