# Supplementary figures and images for: Identification of a Small Molecule That Modifies MglA/SspA Interaction and Impairs Intramacrophage Survival of Francisella tularensis
Source: PLoS One. 2013 Jan 23;8(1):e54498. doi: 10.1371/journal.pone.0054498 (PMC3553074; doi:10.1371/journal.pone.0054498)

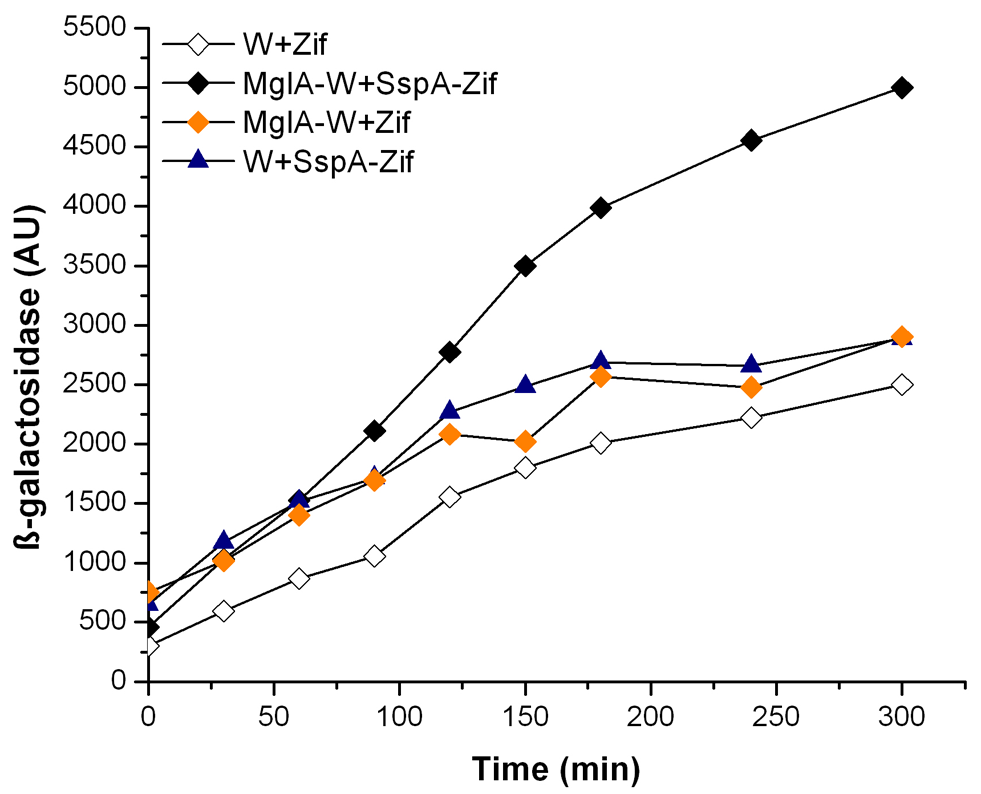

Supplement: Figure S1 — Transcription activation by the interaction between Ft-MglA and Ft-SspA fusion proteins. Different combinations of empty vector and fused proteins were transformed in the E. coli reporter strain AW23 (ΔsspA) and β-galactosidase activity was determined. The plasmid constructs tested were pBR-GP-ω/pACTR-AP-Zif (open diamond, W+Zif), pBR-GP-ω/pACTR-sspA-Zif (blue triangle, W+SspA-Zif), pBR-mglA-ω/pACTR-AP-Zif (orange diamond, MglA-W+Zif), and pBR-mglA-ω/pACTR-sspA-Zif (black diamond). (TIF) [file pone.0054498.s001.tif]

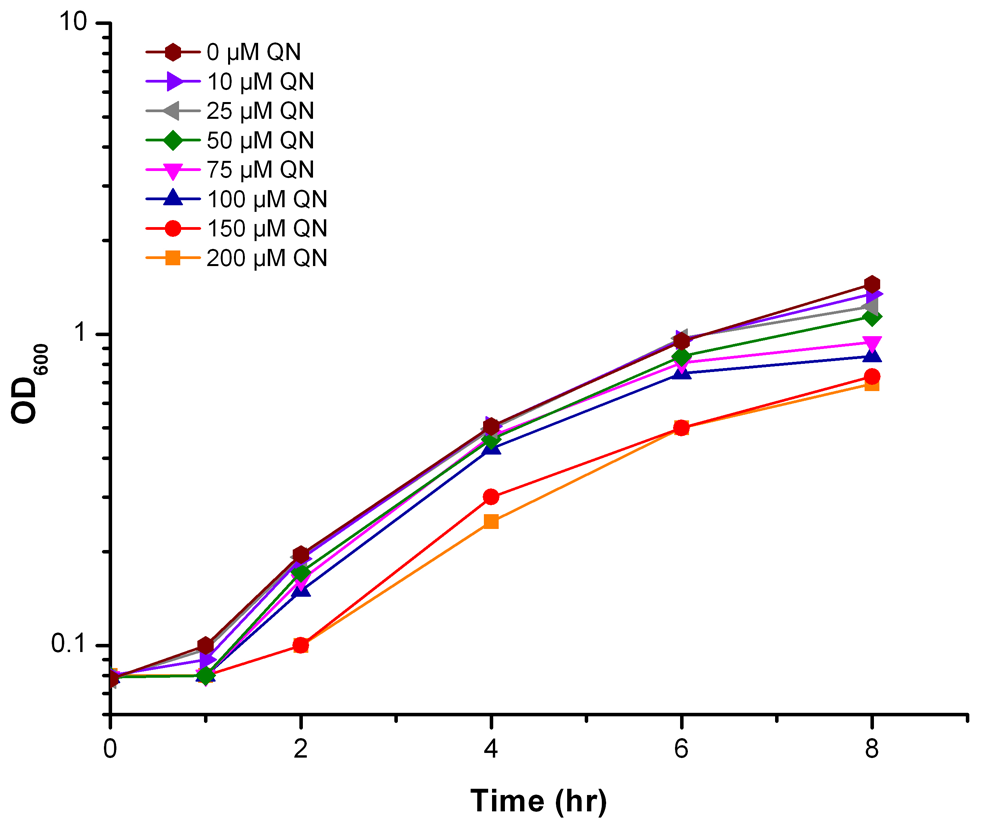

Supplement: Figure S2 — Growth of F. novicida in the presence of quinacrine. The bacterial cells were inoculated in modified TSB containing increasing concentrations of quinacrine (10 to 200 µM). The OD600 was recorded at different time points. The quinacrine concentrations tested were 0 µM (brown circle), 10 µM (right purple triangle), 25 µM (left grey triangle), 50 µM (green diamond), 75 µM (down pink triangle), 100 µM (up blue triangle), 150 µM (red circle) and 200 µM (orange square). (TIF) [file pone.0054498.s002.tif]
